# Pilot Study on the Combination of Different Pre-Treatments with Nanofiltration for Efficiently Restraining Membrane Fouling While Providing High-Quality Drinking Water

**DOI:** 10.3390/membranes11060380

**Published:** 2021-05-24

**Authors:** Yan Chen, Huiping Li, Weihai Pang, Baiqin Zhou, Tian Li, Jian Zhang, Bingzhi Dong

**Affiliations:** 1 School of the Environment and Municipal Administration, Lanzhou Jiaotong University, Lanzhou 730070, China; reneandlhp@163.com (H.L.); zhangjiansanyou@163.com (J.Z.); 2 Key Laboratory of Yellow River Water Environment in Gansu Province, Lanzhou Jiaotong University, Lanzhou 730070, China; 3 State Key Laboratory of Pollution Control and Resource Reuse, College of Environmental Science and Engineering, Tongji University, Shanghai 200092, China; pangweihai@tongji.edu.cn (W.P.); litian001@tongji.edu.cn (T.L.); dbz77@tongji.edu.cn (B.D.); 4 School of Civil and Environmental Engineering, Harbin Institute of Technology (Shenzhen), Shenzhen 518055, China; ziegler.zhou@foxmail.com

**Keywords:** high-quality drinking water, nanofiltration, membrane fouling

## Abstract

Nanofiltration (NF) is a promising post-treatment technology for providing high-quality drinking water. However, membrane fouling remains a challenge to long-term NF in providing high-quality drinking water. Herein, we found that coupling pre-treatments (sand filtration (SF) and ozone–biological activated carbon (O_3_-BAC)) and NF is a potent tactic against membrane fouling while achieving high-quality drinking water. The pilot results showed that using SF+O_3_-BAC pre-treated water as the feed water resulted in a lower but a slowly rising transmembrane pressure (TMP) in NF post-treatment, whereas an opposite observation was found when using SF pre-treated water as the feed water. High-performance size-exclusion chromatography (HPSEC) and three-dimensional excitation–emission matrix (3D-EEM) fluorescence spectroscopy determined that the O_3_-BAC process changed the characteristic of dissolved organic matter (DOM), probably by removing the DOM of lower apparent molecular weight (LMW) and decreasing the biodegradability of water. Moreover, amino acids and tyrosine-like substances which were significantly related to medium and small molecule organics were found as the key foulants to membrane fouling. In addition, the accumulation of powdered activated carbon in O_3_-BAC pre-treated water on the membrane surface could be the key reason protecting the NF membrane from fouling.

## 1. Introduction

Many studies have been conducted to improve drinking water quality by using membrane filtration technologies such as microfiltration (MF), ultrafiltration (UF), nanofiltration (NF), and reverse osmosis (RO). Distinguished by the molecular weight cut-off (MWCO) of the membrane, MF (~10,000 Daltons) and UF (~1000 Daltons) membrane are mainly used to remove insoluble impurities in water, but they show limitations on removing dissolved organic matters. RO (<100 Daltons) membrane intercept nearly all kinds of soluble/insoluble matter, however due to deficient electrolytes, RO-treated water tastes unpleasant to some consumers; hence, people refuse it after prolonged use [1]. NF (~200 Daltons) membranes can effectively remove the majority of harmful substances in water while still retaining some mineral ions [2,3,4,5,6], and due to their larger porous polymeric structures (compared to RO membranes), enable higher volumetric permeate fluxes before discharge, thus exerting great NF potential in manufacturing high-quality drinking water [7,8].

Although NF membranes perform well in advanced water treatment, a large number of practical application results showed that the presence of organic matter, inorganic matter, and microorganisms in the influent water caused serious fouling to the membrane module, resulting in a continuous increase in membrane operating pressure [9,10,11]. Membrane fouling is inevitable and severely restricts the implementation of membrane technology. The major types of fouling in water purification process include the precipitation of inorganic salts, accumulation of suspended particulate matter, and the formation of a biofilm—a mixture of organic matter and microorganisms [12,13]. The precipitation of inorganic salts can be alleviated by the addition of anti-scalants and acid prior to membrane filtration [14]. However, dissolved organic matter (DOM) in feed water is one of the main problems causing membrane fouling, acting as a foremost contributor to membrane fouling. An autopsy of fouled NF membranes in previous studies reported that polysaccharides, protein-like substances, and fulvic acid-like substances were the key membrane foulants [15,16]. Zhao et al. [17] pointed that the complexes formed by metal ions and low MW organic matter were the dominant troublemakers to NF membrane flux decline because of similar complexes’ sizes to the pore diameter of membrane. However, in practice, the organic matter that results in membrane fouling in natural water bodies is extremely complex and no consensus has been drawn over which kind of fractions plays a more important role. Hence, the pre-treatment of feed water is proposed as a method to reduce the concentration of foulants and alter the DOM characteristics. Several proposed means, including slow sand filtration (SSF), ozone–biological activated carbon (O_3_-BAC), ultrafiltration (UF), etc., were reported to have good records in pre-treating feed water [18,19]. However, the quality of raw water matters, because a cost-oriented market requires a trade-off between cost and performance. Sand filtration as the pre-treatment can effectively prevent membrane fouling for raw water which has a decent quality. Although O_3_-BAC has long been proven to be beneficial to remove a wide range of foulants and change the average Stokes radius and apparent molecular weight of DOM [20]. In addition, the UF–NF combined process is flexible to treat various quality of feed water, striking a good balance in producing high-quality water while minimizing operation cost [21,22]. Therefore, which kind of pre-treatment process is optimal for following NF purification deserves deep consideration. Additionally, the contribution to membrane fouling alleviation should be under scrutiny.

The combination of NF membrane technology and running water treatment process of the drinking water treatment plant (DWTP) is both an economical and efficient way for high-quality drinking water manufacture. However, few pilot experiments on the combination of NF membranes and treatment processes in DWTP have been reported. Therefore, effective reference is lacking regarding treatment performance of combined processes and the problem of membrane fouling. The objectives of this study were: (1) to compare the effect on reducing NF membrane fouling by SF and SF+O_3_-BAC pre-treatment processes of a DWTP; and (2) to make sense of the pivotal foulant in membrane fouling. The DOM characteristics of SF-treated water, SF+O_3_-BAC-treated water, and membrane washing solutions were explored by HPSEC and 3D-EEM analysis technology to identify the key foulants and investigate NF membrane fouling mechanisms. In addition, scanning electron microscopy (SEM) and atomic force microscopy (AFM) were used to observe the contamination morphology of the NF membrane surface. After careful analysis of the experimental results, it was found that the NF membrane TMP increased rapidly and suffered biological fouling when SF was used as a pre-treatment for NF. However, the SF+O_3_-BAC pre-treatment process effectively removed the foulants that contributed to the NF membrane fouling. Additionally, also of notice was that powdered activated carbon (PAC) formed a cake layer, which effectively protected the NF from fouling.

## 2. Experiment

### 2.1. Pilot System

**Figure 1:**
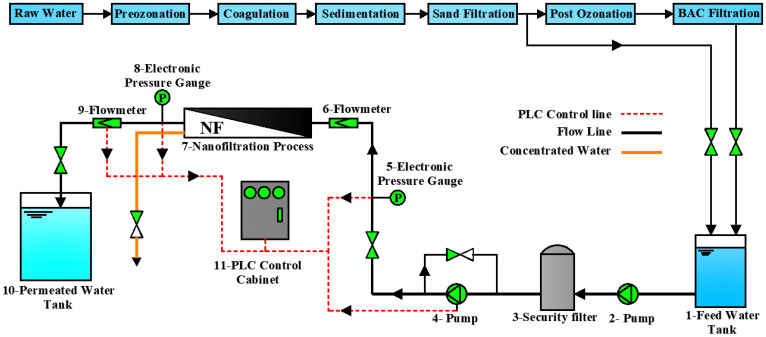
A schematic diagram of the pilot-plant system.

The pilot system for this study was carried out in a DWTP in Suzhou, Jiangsu province, China. A schematic diagram of the pilot is shown in Figure 1. The DWTP process train comprised pre-ozonation, coagulation, sedimentation, sand filtration (SF), post-ozonation, and biological activated carbon (O_3_-BAC). Nanofiltration technology was used to further improve the quality of drinking water. The SF-treated and O_3_-BAC-treated water in DWTP was further treated by the NF pilot system. Both the SF+NF and SF+O_3_-BAC+NF combined treatment processes were studied to explore the water treatment effect and membrane fouling characteristics.

### 2.2. Membrane Cleaning

The virgin NF-90 (DOW Filmtec, Midland, MI) membranes were used in each combined treatment process. During the operation, the NF system was kept at a constant membrane flux of 25 L/(m^2^·h) and the recovery rate was set to 30%. The changes in transmembrane pressure (TMP) data were recorded every other minute by the PLC system during the operation to analyze the membrane fouling rate.

Chemical cleaning in place (CIP) was performed preventively every other month. The fouled membrane was cleaned for one hour with hydrochloric acid solution (pH = 2–3) at 30 °C, followed by one hour of cleaning with sodium hydroxide (pH = 11–12) at 30 °C. The NF washing system operated at full circulation mode; both the concentrated water and permeated water were sent to the water inlet tank. In order to further study the effect of CIP at different stages, the acid and alkaline washing solution was sampled at several time nodes of 20 min, 40 min and 60 min. Additionally, the foulant fractions were analyzed by HPSEC and 3D-EEM fluorescence spectroscopy technology.

### 2.3. Feed Water Quality Parameters

Several water samples, including raw water, SF pre-treated water, SF+O_3_-BAC pre-treated water, and SF+NF- and SF+O_3_-BAC+NF-treated water were sampled to test the concentration and nature of DOM (i.e., DOC, UVA_254_, SUVA and BDOC). Key water quality parameters of the water samples are summarized in Table S1. The reported average concentrations and respective standard deviations were based on at least 15 independent measurements.

### 2.4. Analytical Methods

Turbidity was measured by a turbidimeter (2100N, Hach Company, Loveland, CO, USA). Temperature was recorded by online thermometer. Conductivity and total dissolved solids (TDS) were measured using a conductivity meter (DDS-307A, INESA instrument, Shanghai, China). A UV–Vis spectrophotometer (Evolution 300, Loveland, CO, USA) was used to measure the UV absorbance of the samples at 254 nm (UV_254_). The concentration of DOC was quantified by a total organic carbon (TOC) analyzer (Aurora 1030W, College Station, TX, USA). Specific UV absorbance (SUVA) value was calculated as SUVA (L mg^−1^ m^−1^) = UVA_254_ (cm^−1^)/DOC (mg/L) × 100. The biodegradable organic carbon (BDOC) measurement was performed by quantifying the gross amount of DOC degraded by biological active sand. The active sand was washed 3–5 times by ultra-pure water prior to harvesting for BDOC measurement. Then, 150 mL filtered water sample and 50 g active sand were added into a 250 mL brown iodine measuring flask, and the mixture was incubated at 20 ± 0.5 °C for 10 days. The BDOC calculation formula is shown in Equation (1).

(1) $$\mathrm{BDOC}={\mathrm{DOC}}_{0}-{\mathrm{DOC}}_{10}$$

### 2.5. High-Performance Size-Exclusion Chromatography (HPSEC) Analysis

The molecular weight (MW) distribution of DOM was examined using a Waters-4689 HPSEC instrument (Waters, Milford, MA, USA) combined with an online Sievers-900 TOC analyzer (GE, Boston, MA, USA) and an online 2489 UV/VIS detector (Waters, Milford, MA, USA).

### 2.6. Fluorescence Excitation-Emission Matrix (EEM) Measurements and PARAFAC Modelling

The 3D-EEM fluorescence spectra of water samples were measured with a F7100 Fluorescence Spectrophotometer (Hitachi, Tokyo, Japan). EEM scans were collected by scanning over-emission (Em) wavelengths of 250–550 nm and excitation (Ex) wavelengths of 200–400 nm with a scanning speed at 12,000 nm/min. Both the Ex and Em intervals were set at 2 nm. The fluorescence signal of the pure water (Milli-Q water, Burlington, NJ, USA) was subtracted from the EEMs to remove Raman scatter peaks. The integrated area under the Raman scatter peak (Ex = 350 nm, Em = 381–426 nm) of the pure water was used for Raman unit (RU) normalization [23].

After EEM data pretreatment, a total of 119 fluorescence EEM spectra of water samples (including raw water, feed water, permeated water and membrane washing solutions) were used for parallel factor (PARAFAC) analysis using MATLAB 7.1 based on the DOMFluor toolbox [24]. Finally, a model of five components were identified and validated using split half validation and residual analysis.

### 2.7. Microscopy Imaging

The scanning electron microscopy (SEM) analysis was measured by an SU8020 scanning electron microscope (Hitachi, Tokyo, Japan). The surface morphology of the membranes was analyzed using an atomic force microscope (AFM, FM-NanoviewOpAFM, Suzhou, China).

## 3. Results and Discussion

### 3.1. Effectiveness of the Pre-Treatment on NF

**Figure 2:**
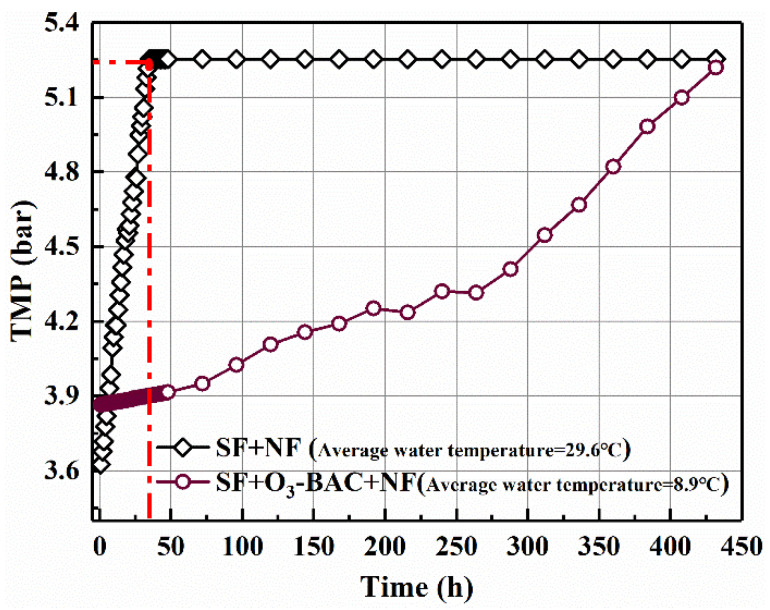
TMP as a function of filtration time of water pre-treated by SF and SF+O_3_-BAC.

The effectiveness of the SF and SF+O_3_-BAC pre-treatment were tested by monitoring the change of the TMP on an NF membrane. Figure 2 shows the changes in the membrane TMP of the NF equipment during continuous operation for treating the SF and SF+O_3_-BAC pre-treated water. It can be seen from the figure that it took only 35 h for the TMP of the NF membrane to increase from the initial 3.63 bar to the ultimate 5.25 bar in the SF+NF combined process, and the upward trend was especially obvious. Due to the NF membrane fouling rate being too fast, the feed pump could not continue to maintain the NF membrane running in a constant flow mode. After 35 h of constant flow operation, the NF membrane began to transform into a constant pressure operation mode, at which time the product water flow rate gradually decreased. In contrast, the TMP increasing rate of the NF membrane in SF+O_3_-BAC+NF process was much slower. Additionally, it took 432 h for the TMP rising from the initial 3.87 bar to 5.25 bar. The running time of NF system was extended 12.34-fold after SF+O_3_-BAC pre-treatment. It is worth noting that the initial TMP of the membrane of the SF+O_3_-BAC+NF process was higher than that of SF+NF, which was due to the water temperature (the average water temperature during the operation of the SF+NF process was 29.6 degrees, while the average water temperature of the SF+O_3_-BAC+NF process was 8.9 degrees). The drop in water temperature reduced the water production performance of the nanofiltration membrane [25,26], thus the inlet water pressure required for the nanofiltration membrane to maintain flux under low temperature conditions was also relatively large. These results indicate that the mitigation effect of SF+O_3_-BAC pre-treatment on NF membrane fouling was far better than that of single SF pre-treatment. Based on this phenomenon, it was necessary to conduct a comparative analysis on the quality of SF- and SF+O_3_-BAC-treated water. The overview of the physical and chemical parameters of the feed waters of the NF installations are shown in Table S1. The average value of turbidity, TDS, conductivity, SUVA, UV_254_, DOC, and BDOC of SF-treated water were 0.143 NTU, 178.06 mg/L, 355.88 μm/S, 1.30 L/(mg∙m), 0.0374 cm^−1^, 2.91 mg/L and 1.69 mg/L, respectively. The water quality of the above indicators treated by O_3_-BAC were 0.119 NTU, 212.00 mg/L, 424.00 μm/S, 0.85 L/(mg∙m), 0.0199 cm^−1^, 2.37 mg/L and 0.95 mg/L, respectively. The SF+O_3_-BAC pre-treatment process significantly cut the content of UV_254_, SUVA and BDOC of the water, but the changes in turbidity, TDS, conductivity, and DOC were not obvious. As a characterization of dissolved organic matter, DOC cannot effectively reflect the membrane fouling potential. This indicates that the effective removal of some specific organic matter (i.e., UVA_254_, SUVA and BDOC) by the O_3_-BAC process may be the key reason for alleviating membrane fouling. In fact, previous studies have shown that the biological properties of organic matter in influent water and the content of unsaturated hydrocarbons were the main factors affecting membrane fouling [27,28]. Additionally, fulvic acid-like substances, polysaccharides, and protein-like substances were also regarded as the key foulants causing NF fouling [16].

Therefore, some specific organic fractions in the feed water are a good indicator to predict membrane biofouling and TMP increasing. In order to better explain the mitigation mechanism of the two pre-treatment processes on NF membrane fouling, HPSEC and EEM analyses were used to further analyze different water samples.

### 3.2. Pre-Treatment Methods and Their Influence on DOM Characteristics

**Figure 3:**
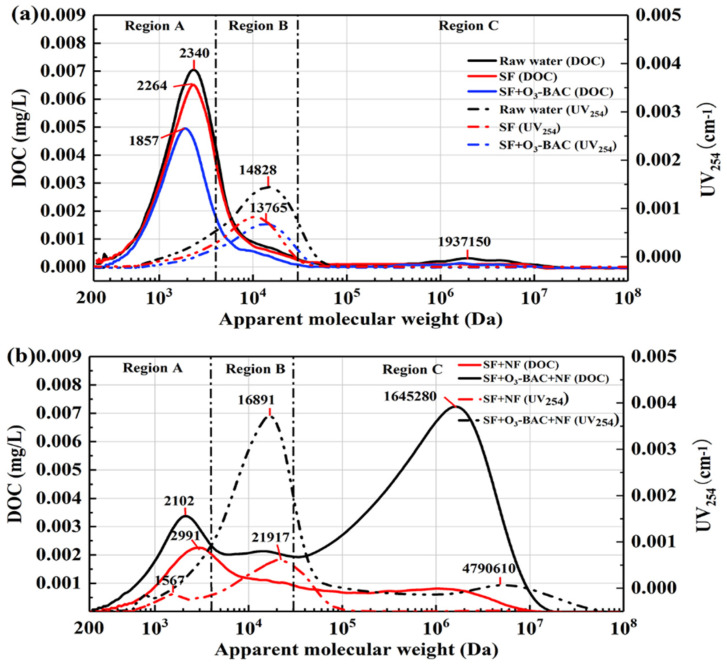
Molecular weight distribution by HPSEC for: (**a**) SF and SF+O_3_-BAC pre-treated water, and (**b**) NF washing solutions (after 30 days of operation).

In order to understand the effect of different pre-treatment on the MW of DOM, HPSEC analysis was performed on specific samples (Figure 3). For in-depth analysis, the DOM fractions were divided into three fractions which were region-A (0.22–4 kDa), region-B (4–30 kDa) and region-C (30–100,000 kDa), according to the size of MW (Table S2). DOC in raw water was mainly distributed in region-A, whereas UV_254_ was mainly distributed in region-B. Therefore, the DOM in region-A was likely to be protein and tyrosine, and region-B was mainly composed of humus and aromatic proteins with a benzene ring structure. The DOM in region-C was proteins and polysaccharides with a macromolecular structure; only a weak peak of DOC was detected by HPSEC.

Comparing the removal performance of SF and SF+O_3_-BAC on organic matter of different regions, the removal of organic matter was substantially better by SF+O_3_-BAC pre-treatment than by SF pre-treatment. The UV_254_ of region-B and DOC of region-C was obviously removed by SF, but the DOC and UV_254_ of region-A were difficult to be removed. This was because the conventional water treatment process using aluminum salt coagulant appeared to have a better removal effect on organic matter with MW greater than 4200 Da, but a poor removal performance on hydrophilic small MW organic matter [29]. After being further treated by an advanced treatment process (O_3_-BAC), we found that both DOC and UV response intensity (especially DOC in 2000–10,000 Da and UV in 4000–10,000 Da) decreased significantly. Ozone resulted in a decomposition of DOM fractions (especially aromatic substances) to lower MW compounds. The reductions in macromolecules (AMW > 4 kDa) of polysaccharides, humic acids, and fulvic acids were mainly attributed to ozonation by O_3_ [29]. In addition, O_3_ improved the biodegradability of water, benefiting the degradation of BDOC by microorganisms on the BAC. Additionally, Table S2 also supports the suggestion that the O_3_-BAC process was far superior to SF in removing BDOC. In addition, the O_3_-BAC process was also found to demonstrate unique performance in removing assimilable organic carbon (AOC) [30,31]. This effectively alleviates the problem of biological contamination of drinking water in the water supply pipeline. Therefore, the mitigation effect of O_3_-BAC on the NF membrane fouling was not only because of reducing the amount of organic matter, but the synergistic effect of O_3_ and BAC characteristically reshaped the nature of NOM as such.

The HPSEC analysis was also performed to reveal the organic fractions of CIP washing solutions of the NF membrane (Figure 3b). The MW characteristics of organic matter in the CIP washing solutions of NF membranes were quite different from the influent water treated by SF or SF+O_3_-BAC. The macromolecular organic matter located in region-C appeared to be an obvious response peak, whereas the response of this response peak of the influent water was extremely weak. In the CIP washing solution of SF+NF, the peak of DOC was mainly located at 2991 Da (region-A), and the UV response peaks were mainly located at 1567 Da (region-A) and 21,917 Da (region-B). In the CIP washing solution of SF+O_3_-BAC+NF, the DOC peaks were mainly located at 2102 Da (region-A) and 1,645,280 Da (region-C), and the UV peaks were located at 16,891 Da (region-B) and 4,790,610 Da (region-C). Additionally, in the SF+O_3_-BAC+NF process, the response intensity of macromolecular organics in the membrane cleaning solution was much higher than that of SF+NF. The above results indicated that macromolecular organics were enriched on the surface of the NF membrane, and these fractions contributed to reversible fouling and were likely eluted. However, the protein-like substances with small molecular structures located in region-A were the key irreversible membrane foulant. In addition, when SF was used as a pre-treatment for NF, the NF membrane was biologically contaminated, which caused the foulants of membrane surface and pore to hardly be eluted.

**Figure 4:**
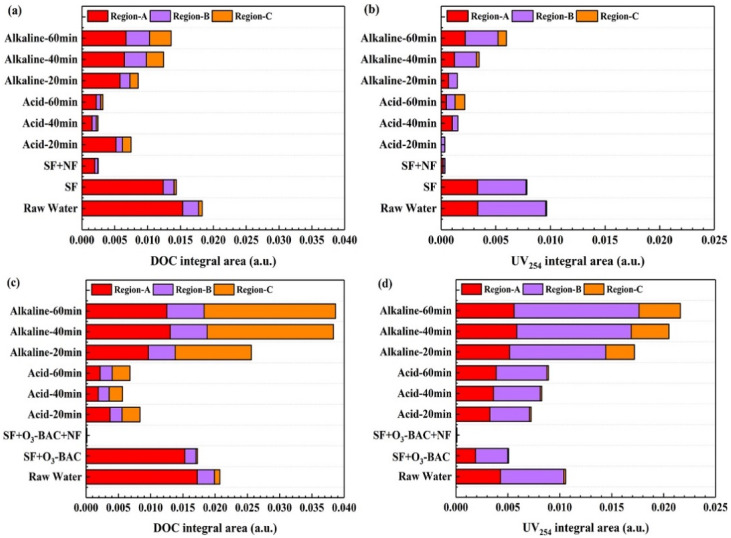
DOC and UVA_254_ integral area of different molecular weight for: (**a**) SF+NF (DOC); (**b**) SF+NF (UVA_254_); (**c**) SF+O_3_-BAC+NF (DOC); and (**d**) SF+O_3_-BAC+NF (UV_254_).

The area integration method was used to deeply explore the MW changes of region-A, region-B, and region-C (Figure 4). In fact, due to the differences in pre-treatment processes, NF membranes demonstrated different removal efficiencies on organic matter. From Figure 4a,b, it can be found that, in SF pre-treated water, the proportions of DOC in region-A, region-B, and region-C were 80%, 19%, and 1%, respectively; the proportions of UV_254_ were 52%, 48% and 0%, respectively. In contrast, in the SF+O_3_-BAC pre-treated water (Figure 4c,d), the proportions of DOC in region-A, region-B, and region-C accounted for 89%, 10%, and 1%, respectively; and the proportions of UV_254_ accounted for 37%, 61% and 2%, respectively. The above results indicated that the SF+O_3_-BAC pre-treatment effectively changed the molecular weight of aromatic organics compared with SF, but the characteristic of DOC did not change significantly. This was attributed to the breakdown of the large aromatic molecules into smaller fragments accelerated the biodegradation of DOM in BAC, where dominant NF membrane foulants were preferentially removed [18]. In addition, in the analysis of the organic fractions of CIP solutions at different stages, the elution effect of acid cleaning was far inferior to the alkaline cleaning against organic matter (especially the macromolecular organic matter of region-C). Additionally, it was also consistent with the physical and chemical indicators of the cleaning solution at different stages (Table S3). This was because acid washing mainly eluted inorganic substances, while alkaline washing had a better elution effect on organic substances. Due to the difference in the content of inorganic substances in the water produced by the SF and BAC pre-treatment processes being relatively small, the organic matter in the alkaline washing solution of the nanofiltration membrane was mainly analyzed here. In the alkaline washing stage, the concentration of membrane foulants of different components in the cleaning solution was positively correlated with the cleaning time. Therefore, this work focused on the analysis of the organic composition of the alkaline washing solution at 60 min. In the SF+NF combined process, the DOC proportions of the membrane alkaline washing solution at 60 min in region-A, region-B, and region-C accounted for 49%, 26%, and 24%, respectively; the proportions of UV_254_ accounted for 37%, 50% and 13%, respectively. In the SF+O_3_-BAC+NF combined process, the DOC proportions accounted for 32%, 15%, and 53%, respectively; and the proportions of UV_254_ accounted for 26%, 56% and 18%, respectively. This showed that when SF was used as a pretreatment for NF, the foulants of the NF membrane were mainly attributed to small molecules, while when SF+O_3_-BAC was used as a pre-treatment, the surface of the foulants which caused NF fouling were mainly organic matter with larger MW. Other studies also suggested that macromolecular organic matter cannot enter membrane pores, thus causing surface pollution, while small molecular substances result in irreversible pollution by entering the membrane pores [32,33].

### 3.3. Identification of Individual Fluorescent Components

**Figure 5:**
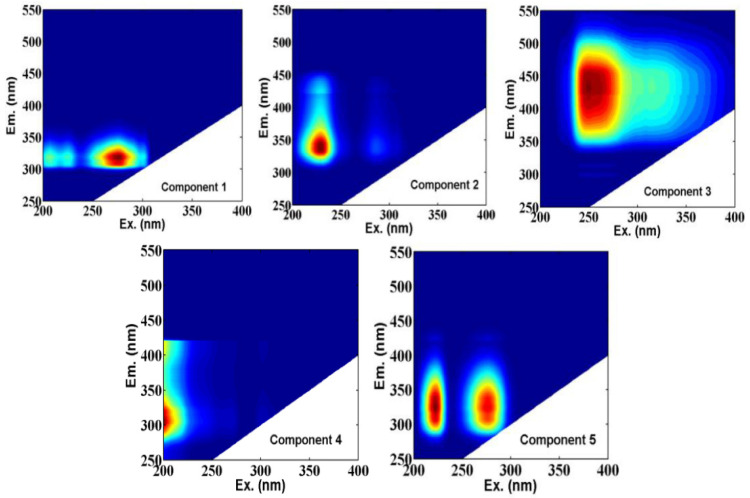
Five fluorescent components identified by the PARAFAC applied to the entire EEM data set of the study. C1: tryptophan-like substances, C2: amino acids, C3: humic-like component, C4: tyrosine-like substances, and C5: tryptophan-like substances.

The three-dimensional excitation–emission matrix (3D-EEM) fluorescence spectroscopy and parallel factor (PARAFAC) analysis are the preferred technologies to identify the key organic substances responsible for membrane fouling and to clarify the underlying fouling mechanisms. Compared with HPSEC analysis, 3D-EEM characterizes the type and concentration of organic matter by the position and intensity of the excitation and emission wavelengths. Differences in fluorescence component plots of the raw water, feed water, and desorbed NF membrane foulants were identified via excitation emission matrix-parallel factor analysis (EEM-PARAFAC). As depicted in Figure 5, five different components were identified for this study. Comparison of previously identified components with the locations of Ex and Em shown in Table S4 indicated that the samples in this study contained tryptophan-like, amino acid, humic-like, as well as protein-like fluorophores. Correlation analysis was used to combine the MW integral area of HPSEC analysis with the F_max_ of PARAFAC. Component 1 (C1) and Component 5 (C5) have previously been ascribed to tryptophan-like substances [34], which appeared strongly correlated with region-A and region-C. Component 2 (C2) was assigned to amino acids [35] which was significantly related to region-B. These substances were mainly derived from algae secretions, phytoplankton, and microbial decomposition. Component 3 (C3) was similar to a humic-like component [36] that was related to region-A and region-B. In this regard, the humic-like component (C3) appeared to be larger in MW and more hydrophobic. Component 4 (C4) was regarded as a tyrosine-like substance [37] with lower MW which was likely to be related to region-A. Compared with our previous studies, only four fluorescent components (C1, C2, C3 and C4) were separated if the samples of NF washing solutions were not included by PARAFAC [38]. In this study, C5 was similar to a combination of components C1 and C2, which was especially detected in the membrane cleaning solution.

The components fate of the raw water, feed water and washing solutions was tracked using their maximum fluorescence intensities (F_max_). It was found that the F_max_ of C1 and C2 accounted for the highest proportion in raw water. Combined with HPSEC analysis, the DOM of raw water mainly consisted of tryptophan, amino acid, and tyrosine-like substances with medium and low molecular structures. After being treated by the SF pre-treatment, the F_max_ values of C1, C2, C3, C4 and C5 were reduced by 8.60%, 20.50%, 27.63%, 25.98% and 55.92%, respectively; and the F_max_ values of the five components were further reduced by 79.34%, 72.26%, 68.40%, 69.27% and 85.01% after being further treated by SF+O_3_-BAC. Compared with the SF pre-treatment, O_3_-BAC apparently improved the removal efficiency of fluorescent organics. The interception of key foulants through high-performance pre-treatment is the main strategy to reduce membrane fouling.

The removal of tryptophan-like aromatic protein (C1) and SMP-like matter by ozonation effectively alleviated membrane pollution [39]. Humic-like components (C3) mainly contributed to reversible fouling [40], and the C3 of the influent water was relatively low. The C4 (with lower MW) was easily degraded by microorganisms; hence, it was recognized as one of the main substances that caused membrane biological fouling.

**Figure 6:**
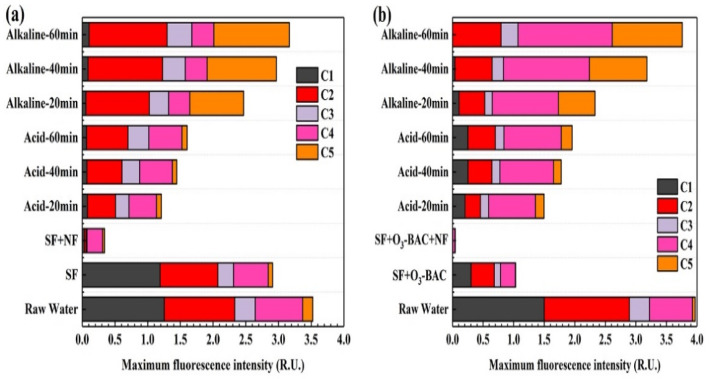
Relative distributions of five different FDOM components in the (**a**) SF+NF combined treatment and (**b**) SF+O_3_-BAC+NF combined treatment.

In addition, combined with the chemical cleaning process of the NF membrane (Figure 6), the proportion of each component in the membrane alkaline washing solution was C2 > C5 > C3 > C4 > C1 in SF+NF combined treatment; and the order of the proportion of each component was C4 > C5 > C2 > C3 > C1 in the SF+O_3_-BAC+NF combined treatment. This seemed to indicate that C2 (amino acids) and C4 (tyrosine-like substances) were the key substances causing NF membrane fouling in this study. The C2 appeared significantly correlated with the organic matter with MW in region-B, which belonged to the fraction of hydrophobic base. The C4, with a small molecular structure, belonged to tyrosine, which was regarded as the component that caused irreversible fouling of membranes and it could not be completely intercepted even by NF treatment (Figure 6). However, the NF membrane appeared to have better performance in terms of the removal of C4 in the SF+O_3_-BAC+NF combined process. It is worth noting that the F_max_ of C5 (tryptophan-like substances) in raw water and influent water (both by SF and SF+O_3_-BAC pretreatment) was very low, but it was especially detected in the cleaning solution. Correlation analysis showed that C5 was significantly correlated with macromolecular organics in region-C. This is consistent with the phenomenon of significant macromolecular peaks in the HPSEC analysis of the cleaning solution. It indicated that C5 only accumulated on the surface of the NF membrane, which was easily cleaned, and would not cause serious membrane fouling.

### 3.4. Surface Morphologies and Properties of the Virgin and Fouled NF Membranes

**Figure 7:**
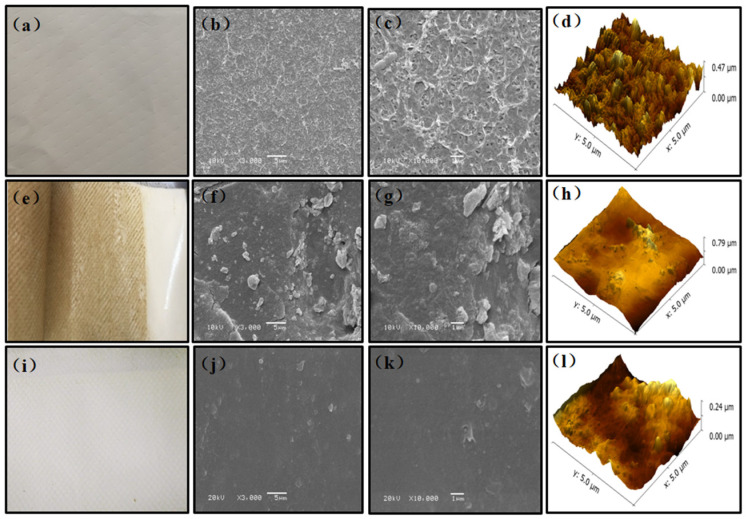
SEM and AFM images of virgin and fouled membranes. (**a**–**d**) Images of virgin NF; (**e**–**h**) images of fouled NF in SF+NF treatment; (**i**–**l**) images of fouled NF in SF+O_3_-BAC+NF treatment.

The surface morphology of the virgin and fouled membranes was also observed by SEM and AFM, and the images are shown in Figure 7. SEM showed that the surface of the virgin NF membrane showed uniform folds, and the same was true for observation through AFM. Compared with the virgin membrane, fouling layers were formed on the NF membrane in the SF+NF combined treatment after one month of filtration. The accumulation of specific organic matter and the formation of biofilms were the main reasons for the above phenomenon.

In SF+NF combined treatment, the biodegradable small molecular organic matter was effectively removed by SF pre-treatment, which caused the NF membrane to be attached by microorganisms to form a thicker biofilm (Figure 7e,f). Once the stable biofilm formed on the surface of the NF membrane, it was difficult to restore the previous flux, and the membrane TMP increased rapidly.

Moreover, no thick pollution layer was formed on the surface of the NF membrane when SF+O_3_-BAC was used as the pre-treatment process of the NF membrane. According to the SEM analysis, the surface of the NF membrane seemed to be covered by a kind of finer substance. Combined with HPSEC and PARAFAC analysis of membrane cleaning solution, these substances were macromolecular substances, which were amino acids, tyrosine-like substances, and tryptophan-like substances. In addition, it also included tiny powdered activated carbon (PAC) from BAC; we detected the accumulation of PAC in the security filter of the membrane system (Figure S1).

This phenomenon was similar to the deposition of powdered activated carbon (PAC) and the formation of a PAC cake layer on an ultrafiltration (UF) membrane surface in the PAC+UF combined process [41,42]. PAC had a double-edged effect on membrane fouling. On the one hand, PAC reduced the amount of DOM deposited on the membrane surface [43,44]. On the other hand, PAC and the deposited DOM have a synergistic effect on membrane fouling when they formed a cake layer together [45]. In this study, PAC was not artificially added, but came from the BAC tank; thus, the PAC concentration in the SF+O_3_-BAC treated water was extremely low. Additionally, the accumulation of PAC was found in the PP cotton of the security filter and the water inlet of the NF membrane after a long period of operation. The adhesion of PAC on the surface of the NF membrane was used as a pre-coating, which intercepted and adsorbed organic matter, and ultimately prevented the formation of biofilms. However, the degree to which membrane fouling was mitigated by the PAC cake layer was not fully confirmed by the research in this article. This research only found that SF+O_3_-BAC pre-treatment showed excellent performance in preventing membrane fouling compared with SF pretreatment. Additionally, this was attributed both to the removal of specific organic matter and the formation of a PAC cake layer by O_3_-BAC. Therefore, this mechanism can be further studied in subsequent experiments.

**Figure 8:**
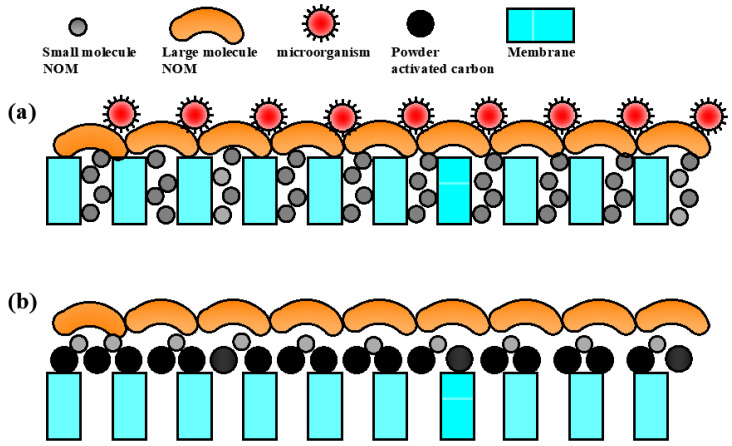
Fouling mechanisms of NF membrane by different pre-treatment. (**a**) SF+NF combined treatment; (**b**) SF+O_3_-BAC+NF combined treatment.

Pre-treatment of fouling substances by simple SF seemed to be limited. O_3_ oxidation and BAC adsorption appeared extremely obvious mitigation effects on NF membrane pollution. In this study, the analysis of influent water difference, organic composition of the membrane cleaning solution, and pollution morphology of the membrane surface were combined to find the fouling reason of nanofiltration membranes and the mechanisms of alleviating membrane fouling by different pre-treatment processes (Figure 8).

For the SF+NF combined treatment process, the NF membrane pores were likely to be blocked by DOM with a low MW DOM of feed water. Additionally, the DOM with larger MW then accumulated on the surface of the membrane, which provided suitable contact sites for microorganisms. Finally, the accumulation of microorganisms led to the formation of a strong biofilm on the surface of the membrane. In fact, the NF membrane was severely polluted by organic matter such as amino acids and tyrosine-like substances at the initial stage in the SF+NF combined process, because it only took 35 h for the TMP of the NF membrane to rise from 3.63 bar to the ultimate 5.25 bar. The aggregation of biodegradable organic matter on the membrane surface provided a good growth environment for microorganisms, which led to the fouling morphology of the NF membrane in the final SEM. For the SF+O_3_-BAC+NF combined treatment process, the NF membrane was mainly contaminated by high MW organic matter, such as tyrosine-like substances and tryptophan-like substances. In addition, from the results of SEM analysis, the PAC from the BAC process also accumulated on the surface of the NF membrane. Although it also caused an increase in membrane TMP, the formed PAC cake layer protected the NF membrane from fouling. PAC had a good adsorption effect against DOM; the formed PAC cake layer reduced the pollution of organic matter to the membrane, and even prevented biological pollution.

## 4. Conclusions

In this study, an NF membrane was combined with the SF and SF+O_3_-BAC treatment processes in a DWTP to conduct pilot studies for high-quality drinking water treatment. The novel findings about NF performance and fouling were presented by investigating the organic matter in feed water and washing solutions using HPSEC and EEM-PARAFAC techniques. SEM and AFM were also used as auxiliary means to analyze the reason for membrane fouling. The main conclusions are as follows:

(1) The SF+NF and SF+O_3_-BAC+NF combined processes appeared to present excellent performance in producing high-quality drinking water, but the combined use of SF+O_3_-BAC was more able to reduce membrane fouling for long term operation.

(2) The DOC concentration in feed water was not a sufficient indicator to measure the potential of membrane fouling, but the number of specific components such as biodegradable organic matter, unsaturated hydrocarbons and humic acid in DOC were the key foulants affecting membrane fouling.

(3) The EEM-PARAFAC combined with HPSEC analysis indicated that the C2 (amino acids) with medium molecular characteristics and C4 (tyrosine-like substances) with small molecular weight were the key substances that caused irreversible membrane fouling; the C3 (humic-like components) and C5 (tryptophan-like substances) mainly contributed to reversible fouling. Compared with SF, the O_3_-BAC process appeared to demonstrate significant removal efficiency of the above-mentioned substances.

(4) The SEM and AFM images showed that a thick foulant layer was formed on the surface of the NF membrane when SF was selected as a pre-treatment process, whereas the NF surface was contaminated slightly when SF+O_3_-BAC was used as a pre-treatment. The deposition of PAC on NF membrane surfaces was one of the key reasons for preventing membrane fouling.

## Supplementary Materials

The following are available online at https://www.mdpi.com/article/10.3390/membranes11060380/s1, Table S1: Test water quality, Table S2: Component analysis of various water constituents based on region A, B and C, Table S3: Analysis of cleaning solutions at different stages in the acid-base washing process, Table S4:Spectral characteristics of the five components identified by PARAFAC analysis and the correlation with the fraction of HPSEC for all samples, Figure S1: The accumulation of powdered activated carbon in the security filter.
