# Supplementary material for: Pilot Study on the Combination of Different Pre-Treatments with Nanofiltration for Efficiently Restraining Membrane Fouling While Providing High-Quality Drinking Water
Source: Membranes (Basel). 2021 May 24;11(6):380. doi: 10.3390/membranes11060380 (PMC8224806; doi:10.3390/membranes11060380)
Supplement: Supplementary file 1 [file membranes-11-00380-s001.zip › membranes-1216341-supplementary.pdf]

Supplementary Material

Pilot Study on the Combination of Different Pre-Treatments with Nanofiltration for Efficiently Restraining Membrane Fouling While Providing High-Quality Drinking Water

Yan Chen [1,2,*,†], Huiping Li [1,2,†], Weihai Pang [3], Baiqin Zhou [4], Jian Zhang [1,2] and Bingzhi Dong [3]

1 School of the environment and municipal administration, Lanzhou Jiaotong University, Lanzhou 730070, China; reneandlhp@163.com (H.L.); zhangjiansanyou@163.com.(J.Z.)
2 Key laboratory of Yellow River Water Environment in Gansu Province, Lanzhou Jiaotong University, Lanzhou 730070, China
3 State Key Laboratory of Pollution Control and Resource Reuse, College of Environmental Science and Engineering, Tongji University, Shanghai 200092, China; pangweihai@tongji.edu.cn (W.P.); dbz77@tongji.edu.cn (B.D.)
4 School of Civil and Environmental Engineering, Harbin Institute of Technology (Shenzhen), Shenzhen 518055, Guang China; ziegler.zhou@foxmail.com
* Correspondence: xiaojingmama@163.com
† These authors contributed equally to this work.

Table S1. Test water quality.

Measurement	Units	Raw water	SF	O_3-BAC	SF+NF	SF+O_3-BAC+NF
Turbidity	NTU	2.4±2.3	0.143±0.023	0.119±0.028	0.055±0.011	0.051±0.010
Conductivity	μm/S	387.6±38.5	355.9±11.1	424.0±24.3	19.34±3.16	10.23±0.8
TDS	mg/L	189.4±19.0	178.1±5.4	212.0±11.9	9.67±1.6	5.15±0.4
UV_{254}	cm^{-1}	0.0537±0.003	0.0374±0.004	0.0199±0.003	0.0016±0.001	0.0008±0.001
DOC	mg/L	3.82±0.30	2.91±0.18	2.37±0.20	0.138±0.137	0.098±0.140
SUVA	$L\cdot mg^{-1} m^{-1}$	1.41±0.09	1.30±0.08	0.85±0.16	1.26±0.811	0.948±0.994
BDOC	mg/L	1.69±0.34	0.95±0.02	0.19±0.07	0.06±0.01	0.01±0.01

Table S2. Component analysis of various water constituents based on region A, B and C[1, 2].

Region	Molecular Weight	Components	Fraction	Functional Groups
Region A	0.22-4kDa	Protein, Tyrosine	Hydrophilic Base	Purines, pyrimidines, low MW alkyl amines
Region B	4-30kDa	Humic, Fulvic Acids	Hydrophobic Acid	Aromatic acids, high MW alkyl mono-, di-carboxylic acids
Region C	30-100000kDa	Proteins, Amino Acids, polysaccharides	Hydrophobic Base	high MW alkyl amines

Table S3. Analysis of cleaning solutions at different stages in the acid-base washing process.

Combined process	Washing time (min)	UV_{254} (cm^{-1})	DOC (mg/L)	Cond. (μm/s)	TDS (mg/L)	pH	Turbidity (NTU)
	Acid 20min	0.0171	1.877	919	458	2.92	0.135
	Acid 40min	0.0202	2.078	729	364	3.10	0.138
	Acid 60min	0.0201	2.694	658	329	3.21	0.104
SF+NF	Alkaline 20min	0.0192	4.958	457	228	11.12	2.850
	Alkaline 40min	0.0301	7.215	451	225	11.03	2.650

	Alkaline 60min	0.0496	11.401	431	215	10.99	2.260
SF+O_3-BAC+NF	Acid 20min	0.0450	5.657	996	483	2.97	0.273
	Acid 40min	0.0556	6.989	880	439	3.04	0.246
	Acid 60min	0.0615	7.823	820	410	3.10	0.269
	Alkaline 20min	0.1227	19.64	775	387	11.43	3.790
	Alkaline 40min	0.1522	29.00	742	371	11.40	4.570
	Alkaline 60min	0.1707	31.92	728	364	11.37	4.600

Table S4. Spectral characteristics of the five components identified by PARAFAC analysis and the correlation with the fraction of HPSEC for all samples.

Component	MW	R^2	Description and probable source
C1	Region-A-DOC	0.558*	tryptophan-like substances
C2	Region-B-UV	0.806**	amino acids
C3	Region-A-UV	0.891**	humic-like components
C4	Region-A-DOC	0.235	tyrosine-like substances
C5	Region-B-DOC	0.972**	tryptophan-like substances

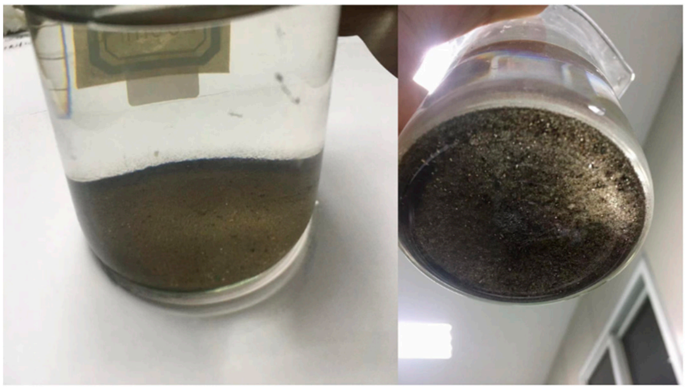

Figure S1. The accumulation of powdered activated carbon in the security filter.

References

1. Brezinski, K., & Gorczyca, B. (2019). An overview of the uses of high performance size exclusion chromatography (HPSEC) in the characterization of natural organic matter (NOM) in potable water, and ion-exchange applications. Chemosphere, 217, 122-139.
2. Teixeira, M. R., & Sousa, V. S. (2013). Fouling of nanofiltration membrane: Effects of NOM molecular weight and microcystins. Desalination, 315, 149-155.
